# Trajectories of women's physical and psychosocial health following obstetric fistula repair in Uganda: a longitudinal study

**DOI:** 10.1111/tmi.13178

**Published:** 2018-11-18

**Authors:** Alison M. El Ayadi, Justus Barageine, Abner Korn, Othman Kakaire, Janet Turan, Susan Obore, Josaphat Byamugisha, Felicia Lester, Hadija Nalubwama, Haruna Mwanje, Vandana Tripathi, Suellen Miller

**Affiliations:** ^1^ Department of Obstetrics, Gynecology and Reproductive Sciences Bixby Center for Global Reproductive Health University of California San Francisco CA USA; ^2^ Department of Obstetrics and Gynaecology Makerere University College of Health Sciences Kampala Uganda; ^3^ Department of Health Care Organization and Policy School of Public Health University of Alabama at Birmingham Birmingham AL USA; ^4^ Urogynaecology Division Mulago National Referral and Teaching Hospital Kampala Uganda; ^5^ EngenderHealth New York NY USA

**Keywords:** vesicovaginal fistula, rectovaginal fistula, obstetric labour complications, urinary incontinence, social stigma, quality of life, fistule vésico–vaginale, fistule recto‐vaginale, complications obstétricales du travail, incontinence urinaire, stigmatisation sociale, qualité de vie

## Abstract

**Objectives:**

To explore trajectories of physical and psychosocial health, and their interrelationship, among women completing fistula repair in Uganda for 1 year post‐surgery.

**Methods:**

We recruited a 60‐woman longitudinal cohort at surgical hospitalisation from Mulago Hospital in Kampala Uganda (Dec 2014–June 2015) and followed them for 1 year. We collected survey data on physical and psychosocial health at surgery and at 3, 6, 9 and 12 months via mobile phone. Fistula characteristics were abstracted from medical records. All participants provided written informed consent. We present univariate analysis and linear regression results.

**Results:**

Across post‐surgical follow‐up, most women reported improvements in physical and psychosocial health, largely within the first 6 months. By 12 months, urinary incontinence had declined from 98% to 33% and general weakness from 33% to 17%, while excellent to good general health rose from 0% to 60%. Reintegration, self‐esteem and quality of life all increased through 6 months and remained stable thereafter. Reported stigma reduced, yet some negative self‐perception remained at 12 months (mean 17.8). Psychosocial health was significantly impacted by the report of physical symptoms; at 12 months, physical symptoms were associated with a 21.9 lower mean reintegration score (95% CI −30.1, −12.4).

**Conclusions:**

Our longitudinal cohort experienced dramatic improvements in physical and psychosocial health after surgery. Continuing fistula‐related symptoms and the substantial differences in psychosocial health by physical symptoms support additional intervention to support women's recovery or more targeted psychosocial support and reintegration services to ensure that those coping with physical or psychosocial challenges are appropriately supported.

## Abstract (fr)

**Objectifs:**

Explorer les trajectoires de la santé physique et psychosociale et leurs relations réciproques chez les femmes ayant subi une réparation de fistule en Ouganda, un an après la chirurgie.

**Méthodes:**

Nous avons recruté une cohorte longitudinale de 60 femmes en hospitalisation de chirurgie à l'hôpital de Mulago, à Kampala en Ouganda (décembre 2014 à juin 2015) et les avons suivies pendant un an. Nous avons collecté des données d'enquête sur la santé physique et psychosociale lors de la chirurgie et à 3, 6, 9 et 12 mois via le téléphone mobile. Les caractéristiques de la fistule ont été extraites des dossiers médicaux. Toutes les participantes ont fourni un consentement éclairé écrit. Nous présentons les résultats de l'analyse univariée et de la régression linéaire.

**Résultats:**

Au cours du suivi postopératoire, la plupart des femmes ont rapporté une amélioration de leur santé physique et psychosociale, principalement au cours des six premiers mois. A 12 mois, l'incontinence urinaire avait diminué de 98% à 33% et la faiblesse générale de 33% à 17%, tandis que l’état de santé général excellent à bon s’élevait de 0% à 60%. La réintégration, l'estime de soi et la qualité de vie ont toutes augmenté au cours des six mois et sont restées stables par la suite. La stigmatisation rapportée a été réduite, mais une certaine perception négative de soi est restée à douze mois (moyenne 17,8). La santé psychosociale était significativement affectée par la déclaration de symptômes physiques; à 12 mois, les symptômes physiques étaient associés à un score de réinsertion moyen inférieur de 21,9 (IC95%: ‐30,1 à ‐12,4)

**Conclusions:**

Notre cohorte longitudinale a connu des améliorations spectaculaires de la santé physique et psychosociale après la chirurgie. La persistance des symptômes liés à la fistule et les différences substantielles en matière de santé psychosociale par symptôme physique soutiennent une intervention supplémentaire pour favoriser le rétablissement des femmes ou des services plus ciblés de soutien psychosocial et de réintégration pour veiller à ce que celles aux prises avec des difficultés physiques ou psychosociales bénéficient d'un soutien approprié.

## Introduction

Prolonged obstructed labour combined with delays in accessing comprehensive emergency obstetric care results in multifaceted physical trauma, often involving multiple organ systems, termed obstructed labour injury complex 1. Although all the conditions associated with obstructed labour injury complex represent significant maternal morbidity, obstetric fistula has been the primary focus of public health programing due to its severe physical, psychological and social sequelae 2, 3, 4, 5, 6. Women with fistula experience constant leakage of urine and/or faeces, are subjected to stigma that frequently results in familial and community isolation 7, 8, and report high rates of depression 6, 9, 10, 11, 12. Accurately estimating the burden of obstetric fistula is a challenge; estimates suggest that as many as 2 million women globally may be affected, with thousands of annual incident cases 13, 14, 15.

Fistula closure rates at hospital discharge range from 65% to 90% 16, 17, 18. Due to the array of physical and psychosocial correlates of fistula, longitudinal studies that follow women beyond the period of short‐term clinical outcomes are of significant interest 3, 19, 20. Studies assessing patient‐focused outcomes of surgery have identified significant short‐term improvements including enhanced overall quality of life 21, psychological status 22, social functioning 23, 24 and economic status 25. However, they also report significant long‐term challenges, including persistent residual incontinence 20, 26, 27, pain and weakness 21, 28, and sexual and fertility complications 5, 21, 29, 30, 31. Lingering physical problems such as these reduce women's ability to resume social roles, making them less likely to consider themselves to have recovered 3, 21, 25, 32, and placing them at greater risk of continued poor mental health 28, 33, 34.

Most longitudinal studies of fistula recovery are limited to a single, short‐term, post‐surgical follow‐up, precluding our ability to understand the process of longer‐term physical and psychosocial recovery. Given the significant physical and psychosocial morbidities experienced by women with fistula, and the lengthy period of time that many women live with fistula prior to accessing treatment, physical and psychosocial recovery is likely a lengthier and more complex process than can be captured at one time point. This study explores women's trajectories of physical recovery and their relationship to psychosocial health following surgical repair of obstetric fistula in Uganda, assessed at four time points over 12 months post‐surgery.

## Methods

We recruited a longitudinal cohort of women accessing fistula surgery at Mulago National Referral and Teaching Hospital in Kampala, Uganda from December 2014 to June 2015 35. Fistula repair within the urogynaecology division is an ongoing surgical service supplemented by several annual fistula repair camps.

Women with obstetric or childbirth‐related iatrogenic fistula were eligible for participation upon completion of initial examination and clearance for surgery if they spoke Luganda or English, resided in a community with cellular telephone coverage, and were capable of providing informed consent. Patients scheduled for surgery were approached and screened for eligibility by research staff, who obtained written informed consent or thumbprint confirmation. Where eligible participants were unable to be approached prior to surgery, they were asked to participate post‐operatively, following sufficient recovery.

Non‐enrolled eligible women included one woman whose fistula resolved through urinary catheterisation and another who refused surgery. Data were captured from the participants prior to surgery (baseline), and 3, 6, 9, and 12 months post‐surgery, and abstracted from medical records. Our baseline questionnaire was administered in person by local research staff in the participant's preferred language and included questions on socio‐demographic characteristics, obstetric history, and physical and psychosocial health measures (Table S1). Aetiology of fistula was inconsistently captured in medical records; we classified fistula aetiology into obstetric *vs*. likely childbirth‐related iatrogenic using criteria proposed by Raasen *et al*. 36. Subsequent surveys included questions on changeable socio‐demographic characteristics, and physical and psychosocial health measures. All four post‐discharge follow‐up surveys were administered over mobile telephone; telephones and airtime were provided to participants. Physical health assessment measures included the Stanford Self‐Rated Health measure for general health 37, the International Consultation on Incontinence Questionnaire Short Form (ICIQ‐SF) 38, self‐reported experience of other fistula‐related symptoms (i.e., faecal incontinence, general body weakness, general pain, pain with urination, vaginal pain, skin irritation, vaginal discharge and difficulty walking) and menstrual irregularity. Psychosocial health assessment included a post‐surgical reintegration success instrument, operationalised as global functioning status specific to women affected by fistula 39, the Hopkins Symptom Checklist (HSC) for depression 40, 41, the WHO Quality of Life (QOL)‐BREF for quality of life 42, 43, the Rosenberg self‐esteem scale 44, a fistula‐related stigma assessment modified from a scale used to measure HIV‐related stigma 45, the Primary Care Post‐traumatic Stress Disorder (PTSD) Screen for trauma 46 and the multidimensional scale of perceived social support 47, 48. We considered a mean HSC score of >1.75 as positive for depressive symptomatology 49, 50. The QOL‐BREF and PTSD screen were not administered at 3 or 9 months to reduce participant response burden. We defined depressive symptomatology using mean HSC score of 1.75 or above, based on commonly used criteria 51, 52. Each interview lasted between 30 and 60 min. Medical record abstraction captured fistula characteristics and surgical outcomes.

Socio‐demographic and obstetric characteristics were described using univariate analyses: means and standard deviations (SD) or medians and interquartile ranges (IQR) for continuous variables and proportions across categorical variables. Physical and psychosocial health measures were standardised to range 0–100 for comparability and are presented similarly. Analyses of psychosocial health separately evaluated domains comprising the WHO QOL‐BREF (domains: overall, physical, environment, social relationships and psychological) and the fistula‐related stigma assessment (domains: negative self‐perception, social isolation, verbal abuse and fear of contagion). We estimated separate linear regression models to understand the relationship between any persistent physical symptoms over time (urinary incontinence, faecal incontinence, general body weakness, pain, pain with urination, vaginal pain, skin irritation, vaginal discharge and difficulty walking) and our psychosocial health measures of reintegration, self‐esteem, depression, quality of life and stigma. All analyses were performed with Stata v14 software (StataCorp, College Station, TX, USA). Differences were considered statistically significant at *P* < 0.05.

The study protocol was approved by the Makerere University College of Health Sciences, School of Medicine Research and Ethics Committee, the Uganda National Council for Science and Technology, and the University of California, San Francisco Human Research Protection Program, Committee on Human Research.

## Results

### Socio‐demographic and obstetric characteristics of study participants

Research staff screened a total of 79 individuals; 62 were eligible, and 60 were enrolled. Retention was 97% at 12 months post‐surgery. Median age at study entry was 28 years (IQR 21–36) (Table 1). Educational attainment varied, but most women had not completed primary school (67%), nor did they work outside the home (58%). Primary sources of financial support were self (30%), husbands (35%) or other individuals (35%), primarily relatives. Household assets varied, with most participants reporting having a radio (58%) and a mobile phone (65%) in the household. Two‐thirds of study participants had living children (65%).

**Table 1:** Socio‐demographic and obstetric characteristics of study participants at baseline (*n* = 60)

Characteristic	*n*	%
Current age in years (median, IQR)	28 (21–36)	
Living situation		
Alone*	13	31.6
Husband*	24	40.0
Parents*	8	13.3
Other*	15	25.1
Any living children	39	65
Educational attainment		
None	10	16.7
Some primary	24	40.0
Completed primary	17	28.3
Any secondary	9	15
Occupation		
None	35	58.3
Vendor/shopkeeper	5	8.3
Farmer	15	25.0
Other	5	8.3
Primary source of financial support		
Self	18	30
Husband	21	35
Other	21	35
Household assets		
Piped water	9	15.0
Flush/pour flush toilet	4	6.7
Electricity	26	43.3
Radio	35	58.3
Television	17	28.3
Mobile phone	39	65
Refrigerator	26	43.3
Age at first birth†	18 (16.5–20)	
Pregnancies before fistula†	1 (0–3)	
Live births before fistula†	1 (0–3)	
Any births since fistula†	7	11.7
Age at fistula†	22.5 (18–31)	
Duration living with fistula		
<1 month	8	13.3
1–3 month	19	31.7
3–12 month	9	13.3
1–2 years	3	5
3–5 years	5	8.3
>5 years	17	28.3
Any ANC for pregnancy resulting in fistula	56	93.3
Health facility delivery for pregnancy resulting in fistula	58	96.7
Infant survived delivery for pregnancy resulting in fistula	17	28.3
Likely aetiology of fistula		
Obstetric	41	68.3
Childbirth‐related iatrogenic	19	31.7

Iqr, interquartile range.

* With or without young children.

† Maximum two participants per each ‘other’ district.

Median age at first birth was 18 years (IQR 17–20). Most women had vesicovaginal fistula only (95%), two women had both vesicovaginal and rectovaginal fistula (3%), and one woman had rectovaginal fistula only (2%). Forty per cent of our participants had developed fistula at their first delivery (40%), with 52% reporting a prior live birth. Seven women (12%) had delivered since fistula development. Time between fistula development and surgery ranged from 2 weeks to 31 years: 13% accessed surgery within 1 month, and 32% between 1 and 3 months; however, 28% lived with fistula for more than 5 years. Most women delivered at a health facility (97%), and only 28% of infants survived. Nearly, one‐third of fistula were classified as likely childbirth‐related iatrogenic (31.7%).

### Physical health across study follow‐up

**Figure 1:**
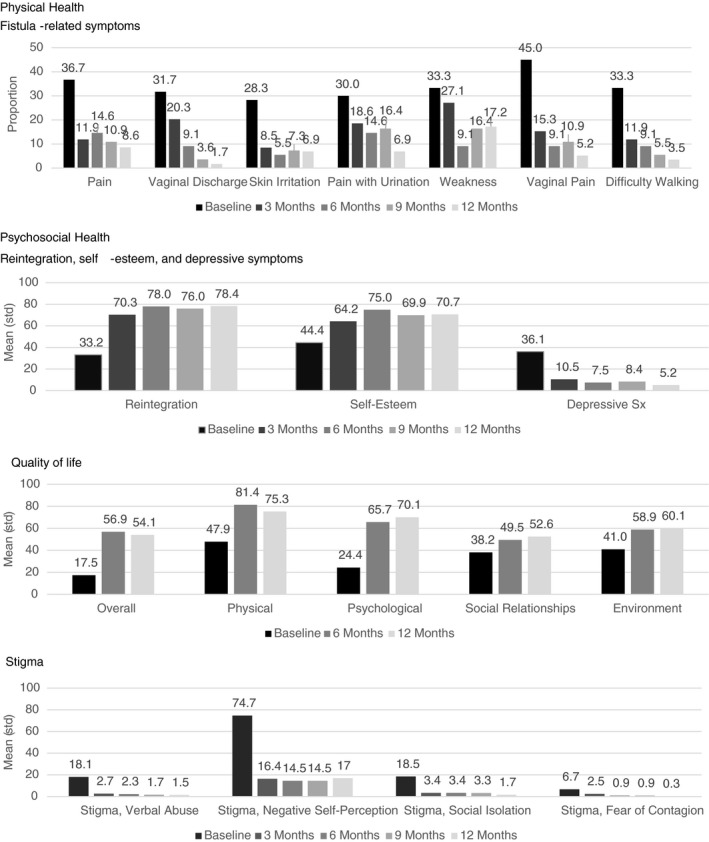
Trajectories of fistula‐related physical symptoms and psychosocial health indicators across 12‐month study follow‐up.

Most women reported fistula‐related symptoms at baseline (Figure 1; Table 2). Self‐report of general health status was poor (82%) or fair (18%). Most women reported urinary incontinence (98%), with 75% reporting it all of the time, and 20% reporting it several times per day. Three women reported faecal incontinence (5%). Nearly half of women reported vaginal pain (45%) and 37% reported general pain. Approximately one‐third reported weakness (33%), difficulty walking (33%), vaginal discharge (32%) or skin irritation (28%). At hospital discharge, leaking was resolved among 37 of 59 women with VVF (63%) and two of three women with RVF. Of the 22 leaking urine at hospital discharge, nine had repair breakdown only, 10 had post‐repair urethral incontinence only and one had both repair breakdown and post‐repair urethral incontinence. The origin of leakage for two participants was not available in their medical record.

**Table 2:** Physical and psychological health across 12‐month study follow‐up

	Baseline	3 months	6 months	9 months	12 months
	*n* = 60	*n* = 59	*n* = 55	*n* = 55	*n* = 58
Physical health					
General health					
Excellent	0 (0)	2 (3.4)	2 (3.6)	0 (0)	0 (0)
Very good	0 (0)	18 (31.5)	30 (54.6)	24 (43.6)	24 (41.4)
Good	0 (0)	15 (25.4)	7 (12.7)	9 (16.4)	16 (27.6)
Fair	11 (18.3)	17 (28.8)	12 (21.8)	18 (32.7)	17 (29.3)
Poor	49 (81.7)	7 (11.9)	4 (7.3)	4 (7.3)	1 (1.7)
Urinary incontinence	59 (98.3)	23 (38.9)	18 (32.7)	20 (36.4)	19 (32.8)
Frequency of urinary incontinence					
Never	1 (1.7)	36 (61)	37 (67.3)	35 (63.6)	39 (67.2)
Once per week or less	0 (0)	1 (1.7)	1 (1.8)	3 (5.5)	5 (8.6)
Two or three times per week	0 (0)	0 (0)	0 (0)	0 (0)	0 (0)
Once per day	2 (3.3)	4 (6.7)	1 (1.8)	2 (3.6)	4 (6.9)
Several times per day	12 (20)	7 (11.9)	6 (10.9)	7 (12.7)	4 (6.9)
All the time	45 (75)	11 (18.6)	10 (18.2)	8 (14.6)	6 (10.3)
Faecal incontinence	3 (5)	1 (1.7)	0 (0)	0 (0)	0 (0)
Menstrual cycle*	*n* = 55	*n* = 54	*n* = 50	*n* = 50	*n* = 53
Regular	18 (32.7)	29 (53.7)	35 (70)	33 (66.6)	32 (60.4)
Irregular	2 (3.6)	2 (3.7)	1 (2)	3 (6)	5 (9.4)
Amenorrheic	35 (63.6)	23 (42.6)	14 (28)	14 (28)	16 (30.2)

Psychosocial health	Mean (SD)	Mean (SD)	Mean (SD)	Mean (SD)	Mean (SD)
Reintegration	33.2 (20.4)	70.4 (27.1)	78.0 (23.7)	75.9 (25.7)	78.4 (19.2)
Self‐esteem	44.4 (15.4)	64.2 (19.0)	72.4 (24.0)	69.9 (20.3)	70.7 (19.8)
Depressive symptoms	36.1 (16.9)	10.5 (13.2)	7.5 (9.6)	8.4 (11.8)	5.2 (7.7)
Quality of life					
Overall	17.5 (10.7)		56.9 (16.5)		54.2 (16.5)
Physical	47.9 (24.2)		81.4 (16.5)		75.3 (13.4)
Environment	41.0 (15.1)		58.9 (11.9)		60.2 (11.2)
Social relationships	38.2 (22.2)		49.5 (22.7)		52.6 (15.9)
Psychological	24.4 (15.1)		65.7 (21.6)		69.0 (17.4)
PTSD screen‐positive *N* (%)	10 (16.7)		1 (1.8)		0 (0)
Stigma					
Negative self‐perception	74.7 (36.2)	16.4 (32.5)	14.5 (29.4)	14.5 (32.9)	17.8 (33.6)
Social isolation	18.5 (27.2)	3.4 (10.1)	3.4 (13.3)	3.3 (10.3)	1.4 (6.1)
Verbal abuse	18.1 (26.5)	2.7 (7.8)	2.3 (7.2)	1.7 (6.4)	1.2 (3.8)
Fear of contagion	6.7 (18.4)	2.5 (8.7)	0.9 (5.0)	0.9 (5.0)	0 (0)
Social support	42.7 (10.0)	45.6 (12.3)	56.1 (20.5)	44.4 (11.6)	55.7 (19.4)

Mean (SD). Improvement is represented by increases in value for reintegration, self‐esteem, and quality of life and decreases in depressive symptoms and stigma. Psychosocial measures are standardised to range 0–100.

* Among age<45 only.

Across the 12‐month study, most women reported improvements in physical health (Table 2). The largest improvement in general health status was observed from baseline to 3 months where the report of general health as excellent, very good or good increased from 0% to 60%. Continued improvement was observed through 6 months (71%). Urinary incontinence, reported by 98% at baseline, was the most prevalent continuing physical symptom. The greatest improvement in urinary incontinence was between baseline and 3 months (39%). Continued reduction in urinary incontinence was reported through 6 months (33%). Among the 19 women reporting persistent incontinence at 12 months, 53% reported it at least once to several or more times per day, and 26% reported it once per week or less. Among those with faecal incontinence at baseline (*n* = 3), the one post‐surgical persistent case resolved by 6 months. Among women aged <45 years, 64% reported amenorrhoea at baseline, which dropped to 43% at 3 months, and to 28% at 6 months.

Other fistula‐related physical symptoms also declined over follow‐up (Figure 1). Large declines were observed from baseline through 3 months in vaginal pain (45–15%), general pain (37–12%), difficulty walking (33–12%) and skin irritation (28–9%). Continued but less pronounced reductions were observed for vaginal pain, general pain and difficulty walking. By 12 months after surgery, 9% reported general pain, 7% skin irritation, 5% reported vaginal pain and 4% difficulty walking. Declines in vaginal discharge were less steep than the other symptoms, dropping from 32% to 20% in the first 3 months following surgery, with only 2% reporting vaginal discharge by 12 months. Pain with urination dropped rapidly between baseline and 3 months (30% to 19%), plateaued at 6 through 9 months (15%) and further decreased by 12 months (7%). General weakness changed minimally from baseline to 3 months (33–27%) and declined more steeply through 6 months (9%), but this gain was not maintained; general weakness at 9 and 12 months was 16% and 17%, respectively. After urinary incontinence, weakness was the second most prevalent fistula‐related symptom at 12 months post‐surgery.

### Reintegration and psychosocial health across study follow‐up

Similarly, improvement was observed across study follow‐up in all psychosocial health indicators of reintegration, self‐esteem, depressive symptoms, quality of life, PTSD, stigma and social support (Figure 1; Table 2). Psychosocial indicators were standardised to range 0–100; unstandardised results are presented in Table S2. Mean reintegration score at baseline was 33.2, increased substantially to 70.4 at 3 months post‐surgery, gained an additional eight percentage points to 78.0 at 6 months and maintained through the end of the study. Self‐esteem at baseline was mean 44.4, increased to 64.2 at 3 months and 72.4 at 6 months, and remained stable thereafter. Mean depressive symptoms also saw the greatest improvement from baseline to 3 months, (36.1–10.5), followed by a small decrease to 5.2 at 12 months. Over the 12 months, depressive symptomatology decreased from 85% to 7% (not shown).

We observed considerable elevations across all quality of life domains from baseline through 6 months post‐surgery, but no further change through 12 months. The greatest mean increases from baseline to 6 months were for overall QOL (17.5–56.9) and the psychological health domain (24.4–65.7). Smaller increases, but still substantial, were observed for social relationships (38.2–49.5) and environment (41.0–58.9) domains. Ten participants (17%) screened positive for PTSD symptoms at baseline, reduced to one (2%) at 6 months and none at 12 months.

At baseline, the highest mean fistula‐related stigma score was observed for the negative self‐perception domain at 74.7 followed by the social isolation and verbal abuse domains (18.5 and 18.1, respectively). Mean score for fear of contagion was the lowest, at 6.7. Substantial declines were observed across all stigma domains during follow‐up, with the largest improvements between baseline and 3 months. Mean stigma score improved by approximately 80% between baseline and 3 months for the domains of verbal abuse, social isolation and negative self‐perception. Continued decline occurred through 12 months for verbal abuse (mean 1.2) and social isolation (mean 1.4). For negative self‐perception, the decline continued through 6 months post‐surgery, but a small uptick occurred at 12 months (mean 17.8).

### Psychosocial health among women with fistula‐related physical symptoms

We assessed the relationship between urinary incontinence or other fistula‐related physical symptoms and indicators of women's psychosocial health (Table 3). Across study follow‐up, self‐report of physical symptoms largely correlated with significantly lower psychosocial health. Mean reintegration score differed by about 20 points at each time point comparing women reporting physical symptoms to those reporting no symptoms. At 12 months, women reporting physical symptoms had a mean reintegration score 21.9 points lower than women reporting no physical symptoms (95% CI: −30.2, −13.5). Self‐esteem among women reporting physical symptoms was also significantly lower: this difference ranged in magnitude from mean 19.3 points lower at 3 months (95% CI −29.3, −9.4) to 21.2 points lower at 12 months (95% CI −30.1, −12.4). Women reporting physical symptoms had significantly higher mean depression at 3, 9 and 12 months by about eight points. At 12 months, women reporting physical symptoms scored on average 7.8 points higher (95% CI 4.3, 11.3) on the standardised depression measure compared to those with no symptoms. Physical symptoms were associated with substantial differences in quality of life domains as well. At 12 months, we observed an approximately 20‐point lower value in overall quality of life (−22.5, 95% CI −28.9, −16.1) and in the physical (−17.1, 95% CI −22.5, −11.7) and psychological domains (−19.1, 95% CI −26.8, −11.4) for women reporting physical symptoms compared to those reporting no symptoms. Slightly lower differences were observed for quality of life domains of social relationships (−13.1, 95% CI −20.7, −5.4) and environment (−13.2, 95% CI −18.0, −8.4). Differences in stigma by the report of physical symptoms were less consistent. Across all time points, large and statistically significant differences in the stigma domain of negative self‐perception were observed, with a mean 22.5 at baseline (95% CI 4.2–40.8) and rising to 34.0 (95% CI 18.8–49.1) at 12 months. Smaller and largely non‐significant differences were identified across symptom categories for the stigma domains social isolation, verbal abuse and fear of contagion. The reduction in difference for social isolation by 12 months was less than that observed for the other domains.

**Table 3:** Unadjusted linear regression models of relationships between urinary incontinence or other fistula‐related physical symptom and psychosocial health across study follow‐up

	3 months		6 months		9 months		12 months	
	B (95% CI)	*P*	B (95% CI)	*P*	B (95% CI)	*P*	B (95% CI)	*P*
Reintegration measure	−24.1 (−38.8, −9.4)	0.0018	−18.2 (−30.2, −6.2)	0.0038	−22.6 (−35.6, −9.5)	0.001	−21.9 (−30.2, −13.5)	<0.0001
Self‐esteem	−19.3 (−29.3, −9.4)	0.0003	−9.1 (−19.8, 1.6)	0.095	−17.9 (−28.2, −7.6)	0.001	−21.2 (−30.1, −12.4)	<0.0001
Depression	10.7 (3.4, 18)	0.0047	2.7 (−2.5, 7.9)	0.3071	8.3 (2.1, 14.5)	0.0098	7.8 (4.3, 11.3)	<0.0001
Quality of life								
Overall			−15.8 (−23.8, −7.8)	0.0002			−22.5 (−28.9, −16.1)	<0.0001
Physical			−17.6 (−25.3, −9.9)	<0.0001			−17.1 (−22.5, −11.7)	<0.0001
Psychological			−17.9 (−28.7, −7.1)	0.0016			−19.1 (−26.8, −11.4)	<0.0001
Social relationships			−13.1 (−25.1, −1.2)	0.0321			−13.1 (−20.7, −5.4)	0.0012
Environment			−3.8 (−10.3, 2.7)	0.2443			−13.2 (−18, −8.4)	<0.0001
Stigma								
Verbal abuse	3.7 (−0.8, 8.2)	0.106	0.3 (−3.6, 4.3)	0.8649	2.2 (−1.4, 5.7)	0.2236	2.3 (0.1, 4.5)	0.0415
Negative self‐perception	22.5 (4.2, 40.8)	0.0169	22.1 (7.1, 37.1)	0.0046	23.5 (6.2, 40.8)	0.0086	34 (18.8, 49.1)	<0.0001
Social isolation	4.9 (−1, 10.8)	0.0989	2.4 (−4.9, 9.7)	0.5122	5.4 (−0.2, 10.9)	0.0568	4.2 (−0.7, 9.1)	0.091
Fear of contagion	3.5 (−1.5, 8.5)	0.1706	−0.9 (−3.6, 1.9)	0.5352	1.5 (−1.3, 4.2)	0.2923	0.6 (−0.6, 1.7)	0.3048

Improvement is represented by increases in value for reintegration, self‐esteem, and quality of life and decreases in depressive symptoms and stigma. At each time point, the values presented reflect the difference between those currently reporting symptoms *vs*. those reporting no symptoms. Psychosocial measures are standardised to range 0–100.

## Discussion

Our study is the first to prospectively assess trajectories of physical and psychosocial recovery in the first year following surgery for obstetric fistula. In this longitudinal cohort of women in Uganda, women experienced dramatic improvements in physical and psychosocial health following surgery. The most substantial gains were typically observed by 3 months with continued improvement through 6 months, after which little change was observed. Further, we quantified the large disparity in psychosocial health between women with persistent physical symptoms compared to those without, identifying significant differences across most indicators.

The most common persistent physical symptoms at 12 months post‐surgery were urinary incontinence (32.8%) and weakness (17.2%). While the 12‐month values represent a substantial reduction from baseline, the proportion of women with persistent symptoms is nonetheless concerning. Our findings on urinary incontinence were on the higher end of those reported in other studies reporting persistent incontinence rates of 16–35% 13, 18, 32, 53, 54, 55. Our identification of non‐incontinence‐related persistent symptoms is also not unique; in a qualitative study of women repaired for fistula in Ethiopia, Donnelley *et al*. 28 reported that 40% of women reported physical health problems following surgery irrespective of their repair status. The substantial and significant differences in level of reintegration comparing women with persistent physical symptoms and those without support additional intervention to resolve physical symptoms in order to support women's recovery or more targeted psychosocial support and reintegration services to ensure that those coping with physical or psychosocial challenges are appropriately supported.

Our findings demonstrating dramatic improvement in quality of life following surgery are consistent with other reports 20, 24, 31 although several interesting differences in mean value and changes over time across setting are notable in two studies in India and Nigeria also using the WHO QOL‐BREF that followed women through 6 months (Nigeria) and median 26 months (India) after surgery 56, 57. The lowest pre‐operative QOL score for all domains except social relationships and the largest improvements from the pre‐ to the post‐operative period were in India. In India and Nigeria, significant improvements were noted in the physical, psychosocial and social domains but no change was observed for the environment domain. The largest improvements in Uganda and India were observed in the physical domain. Further comparison with population‐based samples of healthy women of reproductive age would help to place these data into context. Additional research on the contextual factors responsible for the differences identified in overall experience and change across time could help inform efforts to improve recovery trajectories across various cultural contexts.

We found substantial reductions across the dimensions of fistula‐related stigma for the 12‐month period, with the steepest declines observed from baseline through 3 months. In this cohort, negative self‐perception was the most severe type of stigma experienced across all time points. Compared to negative self‐perception, the relatively lower report of stigma domains representing enacted stigma (verbal abuse, social isolation and fear of contagion) may be partially explained by nearly half of our cohort achieving surgery within 3 months of fistula development. Our finding of persistent stigma is consistent with one qualitative study of Ethiopian women's post‐surgical quality of life, where persistent internalised and enacted stigma were reported at mean 1.5 years following surgery irrespective of surgical outcome 28. Literature from other stigmatised conditions describes a complex relationship between perceived community stigma and internalised stigma 58 and supports a strong relationship between internalised stigma and depression and other poor health outcomes 59, 60, 61, highlighting the need for psychosocial intervention at multiple levels to reduce stigma experiences and associated consequences 62, 63.

The substantial differences observed in psychosocial outcomes comparing women with physical symptoms to those without highlight the need for identification of strategies to treat or better manage post‐surgical fistula‐related symptoms. Our results suggest that the greatest overall physical health benefits occur during the first 6 months following surgery, with concurrent psychosocial health improvement. The stagnation in physical and psychosocial health indicators that we observed at about 6 months is consistent with qualitative work conducted Kenya by Khisa *et al*. 64. Khisa describes a ‘miracle phase’ in the immediate post‐surgical period among women with successful repair. While some of the stagnation in trend may be due to floor and ceiling effects, our results may be also be influenced by individual experiences not explored here including fistula‐related characteristics or experiences, sexual relationships, concerns around achieving or preventing pregnancy and exposure to violence. Other factors, such as poverty, lack of empowerment and lack of social support, may also structure health status, and thus, our findings may represent physical and psychosocial health status comparable to similar women of reproductive age in this setting who did not develop fistula. Subsequent work should explore the contributions of other factors, and development of interventions to improve recovery will benefit from the inclusion of theory‐based conceptual models for recovery from mental health diagnoses suggest areas for counselling foci that are particularly salient for women recovering from fistula, based on our findings here and other literature 65.

We recognise several limitations to this study. Our sample size was based on feasibility; thus, our results should be validated in larger samples. Despite our small sample, our rates of participation were high, and our cohort was representative of women accessing fistula repair at Mulago Hospital during the recruitment period. Both similarities and important differences in women's experiences of fistula and recovery are observed in the literature, and thus, a nuanced assessment of the contextual factors contributing to these differences across countries will help establish the generalisability of our results. In particular, many women in our cohort rapidly accessed surgery and nearly thirty per cent of infants survived the labour resulting in the fistula, suggesting improved care access for this cohort compared to some other studies. Our sample included participants with childbirth‐related fistula due to pressure necrosis from obstructed labour and iatrogenic aetiologies; however, designation of likely iatrogenic among this sample was retrospectively assigned using medical record criteria 36. Our findings can be considered generally representative of women with obstetric fistula, but further research exploring differences in recovery trajectories by aetiology is of interest, particularly as iatrogenic female genital fistula appears to be increasing 66. Our study is both strengthened and limited by the length of follow‐up; our focus on the 12 months following surgery expands the length of follow‐up conducted in many other studies, yet may be inadequate to understand the rates and consequences of fistula recurrence and adverse perinatal outcome over a woman's reproductive life, both of which have been found to be high among women receiving surgical repair for obstetric fistula 67.

Reducing the impact of fistula‐related maternal morbidity on the health of women requires their engagement in the full continuum of fistula‐related management. Prevention is key, and improving women's timely access to surgical repair of fistula is critical. Yet limiting attention to women recovering from fistula in the immediate post‐surgical period is inadequate. Intervention programming to support the spectrum of women's physical and psychosocial needs during their recovery from fistula may result in improved outcomes after repair. Efforts must also be made to maintain women's engagement with health systems and to improve the responsiveness of health systems to women's needs following surgery to protect maternal health and well‐being.

## Funding

The study was funded by the Eunice Kennedy Shriver National Institute of Child Health and Human Development of the United States National Institutes of Health (NICHD; Project number R21HD075008), the Fistula Foundation and from the United States Agency for International Development (USAID) via the Fistula Care *Plus* Project, administered by EngenderHealth (cooperative agreement AID‐OAA‐A14‐00013). The opinions expressed are those of the authors and do not necessarily reflect the views of USAID, or the United States Government. Continuing analytical work was funded by NICHD (Project number K99HD086232).

## Ethics approval

The study protocol was approved by the Makerere University College of Health Sciences, School of Medicine Research and Ethics Committee (#2014‐052, Apr 2014), the Uganda National Council for Science and Technology (#154/212/01, June 2015), and the University of California, San Francisco Human Research Protection Program, Committee on Human Research (#12‐09573, Oct 2012).

## Supporting information


**Table S1**. Description of physical and psychosocial health measures.
**Table S2**. Psychosocial health indicators across study 12‐month study follow‐up, Unstandardised.
