# Supplementary material for: Trajectories of women's physical and psychosocial health following obstetric fistula repair in Uganda: a longitudinal study
Source: Trop Med Int Health. 2018 Nov 18;24(1):53–64. doi: 10.1111/tmi.13178 (PMC6324987; doi:10.1111/tmi.13178)
Supplement: Supplementary file 1 — Table S1. Description of physical and psychosocial health measures. Table S2. Psychosocial health indicators across study 12‐month study follow‐up, Unstandardised. [file TMI-24-53-s001.docx]

Supplemental Table 1. Description of Physical and Psychosocial Health Measures

| **Concept** | **Validated Measure** | **Items** | **Definition** | **Range** |
| --- | --- | --- | --- | --- |
| **Physical Health** |  |  |  |  |
| General Health | Stanford Self-Rated Health Measure | 1 | Subjective evaluation of individual general health. | 1-5 |
| Urinary Incontinence | International Consultation on Incontinence Questionnaire Short Form (ISIQ-SF) | 4 | Assessment of urinary incontinence frequency, amount of leakage, and overall impact of urinary incontinence on quality of life. | 0-21 |
| Faecal Incontinence | Modified from ISIQ-SF. | 2 | Assessment of faecal incontinence frequency and overall impact of faecal incontinence on quality of life. | 0-15 |
| Weakness | No | 1 | Self-report of current presence or absence of symptom. | - |
| General Pain | No | 1 | Self-report of current presence or absence of symptom. | - |
| Pain with Urination | No | 1 | Self-report of current presence or absence of symptom. | - |
| Vaginal Pain | No | 1 | Self-report of current presence or absence of symptom. | - |
| Skin Irritation | No | 1 | Self-report of current presence or absence of symptom. | - |
| Foul-smelling Vaginal  Discharge | No | 1 | Self-report of current presence or absence of symptom. | - |
| Difficulty Walking | No | 1 | Self-report of current presence or absence of symptom. | - |
| Menstrual Irregularity | No | 1 | Self-report of current menustrual cycle: regular, irregular or amenorrheic/not menstruating. | - |
| **Psychosocial Health** |  |  |  |  |
| Reintegration | Post-surgical Reintegration Success Instrument^1^ | 18 | Global functioning status specific to women affected by fistula, characterized by four sub-factors of mobility and social engagement, meeting family needs, comfort with relationships, and general life satisfaction. | 0-100 |
| Depression | Hopkins Symptoms Checklist^2,3^ | 15 | Self-report symptom inventory for detection of depressive disorder. | 0-4^1,2^ |
| Quality of Life | World Health Organization Quality of Life BREF (WHO QOL BREF)^4,5^ | 26 | Core set of items agreed upon for cross-cultural assessment of quality of life, representing the 24 facets of QOL, organized into 4 domains (below). | 0-100 |
| Overall | WHO QOL BREF^4,5^ | 2 | Overall perception of quality of life and quality of health. | 0-100 |
| Physical | WHO QOL BREF, domain ^4,5^ | 7 | Activities of daily living, dependence on medical care, energy and fatigue, mobility, pain and discomfort, sleep and rest, work capacity. | 0-100 |
| Psychological | WHO QOL BREF, domain ^4,5^ | 6 | Bodily image and appearance, negative and positive feelings, self-esteem, spiritualty, thinking learning, memory and concentration. | 0-100 |
| Social Relationships | WHO QOL BREF, domain ^4,5^ | 3 | Personal relationships, social support, sexual activity. | 0-100 |
| Environment | WHO QOL BREF, domain ^4,5^ | 8 | Financial resources, freedom, physical safety and security, accessibility and quality of health and social care, home environment, opportunities for acquiring new information and skills, participation in and opportunities for recreation activities, physical environment, and transport. | 0-100 |
| Self-esteem | Rosenberg Self-Esteem Scale^6^ | 10 | Subjective emotional evaluation of one's self-worth. | 0-30^1,3^ |
| Fistula-related stigma assessment | Modified from HIV/AIDS Stigma Instrument - People Living with HIV/AIDS (HASI-P) ^7^ | 18 | Perceived devaluation of individuals with fistula, across four domains: verbal abuse, negative self-perception, social isolation, and fear of contagion. | 0-100 |
| Verbal abuse | Modified from HASI-P, domain^7^ | 8 | Behavior or actions intended to harm the person with fistula, such as insult or ridicule | 0-100 |
| Negative self-  perception | Modified from HASI-P, domain^7^ | 2 | Negative view of oneself based on fistula status, including feeling worthless or ashamed. | 0-100 |
| Social isolation | Modified from HASI-P, domain^7^ | 6 | Intentional exclusion from social events due to fistula status. | 0-100 |
| Fear of contagion | Modified from HASI-P, domain^7^ | 2 | Avoidance of person with fistula due to fear of contagion. | 0-100 |
| Post-Traumatic Stress Disorder | Primary Care Post-Traumatic Stress Disorder Screen^8^ | 4 | Any lifetime experience so upsetting it resulted in past month: nightmares, avoidance of thoughts or situations, anxiety, or detachment. | - |
| Social Support | Multidimensional Scale of Perceived Social Support^9,10^ | 12 | Subjectively assessed adequacy of social support from family, friends, and significant other. | 1-5^1,2^ |

*^1^Standardized to range 0-100 for current analysis for comparability across measures; ^2^Hopkins Symptom Checklist and Multidimensional Scale of Perceived Social Support evaluated as mean score across items.*

**Supplemental Table 2. Psychosocial health indicators across study twelve-month study follow-up, Unstandardized**

|  | Baseline | 3 Months | 6 Months | 9 Months | 12 Months |
| --- | --- | --- | --- | --- | --- |
|  | n=60 | n=59 | n=55 | n=55 | n=58 |
| Reintegration | 38·8 (18·5) | 70·5 (25·5) | 80·0 (20·9) | 75·7 (24·2) | 83·1 (14·4) |
| Self-Esteem | 13·3 (4·6) | 19·2 (5·7) | 22·5 (6·0) | 20·9 (6·1) | 21·3 (5·8) |
| Depressive symptoms | 2·4 (0·7) | 1·4 (0·5) | 1·3 (0·4) | 1·3 (0·5) | 1·2 (0·3) |
| Quality of Life |  |  |  |  |  |
| Overall | 17·5 (10·7) |  | 56·9 (16·5) |  | 55·3 (16·4) |
| Physical | 47·9 (24·2) |  | 81·4 (16·5) |  | 77·4 (11·4) |
| Environment | 41·0 (15·1) |  | 58·9 (11·9) |  | 61·1 (10·6) |
| Social Relationships | 38·2 (22·2) |  | 49·5 (22·7) |  | 52·3 (14·6) |
| Psychological | 24·4 (15·1) |  | 65·7 (21·6) |  | 70·3 (15·9) |
| PTSD Screen Positive N (%) | 10 (16·7) |  | 1 (1·8) |  | 0 (0) |
| Stigma |  |  |  |  |  |
| Negative Self-Perception | 74·7 (36·2) | 16·4 (32·5) | 14·5 (29·4) | 14·5 (32·9) | 17·8 (33·6) |
| Social Isolation | 18·5 (27·2) | 3·4 (10·1) | 3·4 (13·3) | 3·3 (10·3) | 1·4 (6·1) |
| Verbal Abuse | 18·1 (26·5) | 2·7 (7·8) | 2·3 (7·2) | 1·7 (6·4) | 1·2 (3·8) |
| Fear of Contagion | 6·7 (18·4) | 2·5 (8·7) | 0·9 (5·0) | 0·9 (5·0) | 0 (0) |
| Social Support | 42·7 (10·0) | 45·6 (12·3) | 56·1 (20·5) | 44·4 (11·6) | 55·7 (19·4) |

*Mean (SD). Improvement is represented by increases in value for reintegration, self-esteem, and quality of life and decreases in depressive symptoms and stigma.*

**References**

1. El Ayadi A, Byamugisha J, Obore S, et al. Development and preliminary validation of a post-fistula repair reintegration instrument among Ugandan women. *Reproductive Health Journal* 2017; **14**(1): 109.

2. Bolton P, Wilk CM, Ndogoni L. Assessment of depression prevalence in rural Uganda using symptom and function criteria. *Social psychiatry and psychiatric epidemiology* 2004; **39**(6): 442-7.

3. Derogatis LR, Lipman RS, Rickels K, Uhlenhuth EH, Covi L. The Hopkins Symptom Checklist (HSCL): A self-report symptom inventory. *Behavioral Science* 1974; **19**(1): 1-15.

4. Martin F, Russell S, Seeley J. The WHOQOL BREF questionnaire in Luganda: Validation with a sample including people living with HIV in Uganda. Working Paper 46. Norwich, United Kingdom: School of International Development, University of East Anglia, 2013.

5. Webster J, Nicholas C, Velacott C, Cridland N, Fawcett L. Validation of the WHOQOL-BREF among women following childbirth. *Aust N Z J Obstet Gynaecol* 2010; **50**(2): 132-7.

6. Rosenberg M. Society and the adolescent self-image. Princeton, NJ: Princeton University Press; 1965.

7. Holzemer WL, Uys LR, Chirwa ML, et al. Validation of the HIV/AIDS Stigma Instrument - PLWA (HASI-P). *AIDS Care* 2007; **19**(8): 1002-12.

8. Prins A, Ouimette P, Kimerling R, et al. The primary care PTSD screen (PC_PTSD): development and operating characteristics. *Primary Care Psychiatry* 2003; **9**: 9-14.

9. Zimet GD, Powell SS, Farley GK, Werkman S, Berkoff KA. Psychometric characteristics of the Multidimensional Scale of Perceived Social Support. *Journal of personality assessment* 1990; **55**(3-4): 610-7.

10. Nakigudde J, Musisi S, Ehnvall A, Airaksinen E, Agren H. Adaptation of the multidimensional scale of perceived social support in a Ugandan setting. *Afr Health Sci* 2009; **9 Suppl 1**: S35-41.
